# Low Back Pain—Behavior Correction by Providing Haptic Feedbacks: A Preliminary Investigation

**DOI:** 10.3390/s21217158

**Published:** 2021-10-28

**Authors:** Andrea Ferrone, Astrid García Patiño, Carlo Menon

**Affiliations:** 1 Menrva Research Group, Schools of Mechatronic Systems & Engineering Science, Simon Fraser University, Metro Vancouver, BC V5A 1S6, Canada; andrea_ferrone@sfu.ca (A.F.); agarciap@sfu.ca (A.G.P.); 2 Biomedical and Mobile Health Technology Lab, Department of Health Sciences and Technology, ETH Zurich, Lengghalde 5, 8008 Zurich, Switzerland

**Keywords:** haptic feedback, inertial measurement unit, low back pain, monitoring, nurses, wearable device

## Abstract

The activities performed by nurses in their daily activities involve frequent forward bending and awkward back postures. These movements contribute to the prevalence and development of low back pain (LBP). In previous studies, it has been shown that modifying their posture by education and training in proper lifting techniques decreases the prevalence of LBP. However, this education and training needs to be implemented daily. Hence, implementing the use of a wearable device to monitor the back posture with haptic feedback would be of importance to prevent LBP. This paper proposes a wearable device to monitor the back posture of the user and provide feedback when the participant is performing a possible hurtful movement. In this study, a group of participants was asked to wear the device while performing three of the most common activities performed by nurses. The study was divided into three sessions: In the first session, the participants performed the activities without feedback (baseline). During the second session, the participants received feedback from the wearable device (training) while performing the three tasks. Finally, for the third session, the participants performed the three tasks again, but the haptic feedback was turned off (validation). We found an improvement in the posture of more than 40% for the pitch (lateral bending) and roll (forward/backward bending) axes and 7% for the yaw (twisting) axis when comparing to the results from session 1 and session 2. The comparison between session 1 and session 3 showed an overall improvement of more than 50% for the pitch (lateral bending) and roll (forward/backward bending) axes and more than 20% for the yaw axis. These results hinted at the impact of the haptic feedback on the participants to correct their posture.

## 1. Introduction

Low back pain (LBP) is ranked sixth in terms of the overall burden of disease worldwide [1]. The World Health Organization recently stated that “musculoskeletal conditions are the leading contributor to disability worldwide, with low back pain being the single leading cause of disability globally” [2]. While little is known about the development of LBP between adolescence and early professional life [3], there is a high development of LBP in adults. Jaromi et al. estimated that 70% to 85% of adults experienced back pain at least once in their lifetime [4]. LBP appears equally in women and men between the ages of 30 and 50 years [4,5,6,7]. The higher prevalence rate of LBP is found among health workers [5,8,9]. It has been proven that among health workers, nurses are at higher risk of having LBP or spine injuries [10,11,12,13]. This could be explained by the fact that nurses are daily involved in activities such as patient handling and repositioning, as well as many tasks involving bending, twisting, pushing, and pulling [3,8,11,12,13,14]. The topic of LBP is extremely difficult to study since the causes for LBP are unclear and are rarely directly linked to a definitive issue or condition [7,9,10,15].

A series of techniques and methods have been proposed in an effort to reduce the prevalence of LBP, such as physical treatments, exercise therapy, manual therapy, education, pharmacological procedures, and invasive procedures [16].

Several studies suggested that education about back posture and proper lifting techniques is effective in the treatment and prevention of LBP [4,17,18,19,20]. Considering the relation between back posture and LBP, it raises the importance of monitoring trunk movements in real-time to provide continuous feedback.

Continuous feedback could be adopted as a behavior change device or app (e.g., Fitbit, Spark, Nike Fuelband), which will give feedback/reward to reinforce positive habits based on performances recorded [21].

Nowadays, this approach is possible thanks to technologies developed for the field of motion tracking that are wearable and less obtrusive [22]. There are some wearable technologies available in the market for back posture monitoring, such as BackTone posture corrector, Kinetic Reflex Smart Wearable, Prana wearable device, Alex wearable device, Upright Posture Training Device, and Life-booster [23,24,25,26,27,28]. Most of them are based on Inertial Measurement Units (IMU) and are focused on a single task.

Upright, BackTone, and Prana wearable posture correctors focus on upper back and shoulder slouching [23,25,27]. The Alex wearable device detects the posture of the neck [26]. The Kinetic Reflex Smart Wearable device detects squatting vs. forward bending movements [24]. More complex systems such as Life-booster implement several measurements units distributed on the body and incorporate camera recordings [28]. This system can detect a wide range of movements and postures. However, the Life-booster system has practical limitations such as body encumbrance and wearability. Furthermore, cameras can be adopted only in closed or limited areas where there are no limitations or concerns of privacy. All of the aforementioned devices provide haptic feedback to the user when a posture/movement that is considered incorrect is detected.

Despite the variety of back posture monitoring devices available in the market, the need for accurate back posture monitoring during the most common activities of nurses (e.g., patient transfer, boosting patient in bed, and reaching task) remains unfulfilled [3,9,10,29]. In this study, we introduce a wearable back posture monitoring device and study its impact to avoid awkward postures. The wearable device was tested with multiple participants performing three of the most common activities performed by nurses. The performance and improvement of the participants before, during, and after the haptic feedback using the wearable device was investigated.

## 2. Methods

### 2.1. Participants

Nine healthy females (age: 26.0 ± 11.2 years, height: 1.58 ± 0.06 m, weight: 56.7 ± 7.1 kg) participated in the study and provided informed written consent. Exclusion criteria included upper extremity musculoskeletal injury and/or existence of low back pain conditions. All participants were recruited from Simon Fraser University Campus. None of the participants had experience or training for proper lifting practices, patient handling, or other suggested manual material handling. The recruitment of more participants and data collection was not possible due to COVID-19 restrictions by the BC Health Authority and Simon Fraser University. This study was approved by the Research Ethics Board at Simon Fraser University.

### 2.2. Experimental Setup

**Figure 1:**
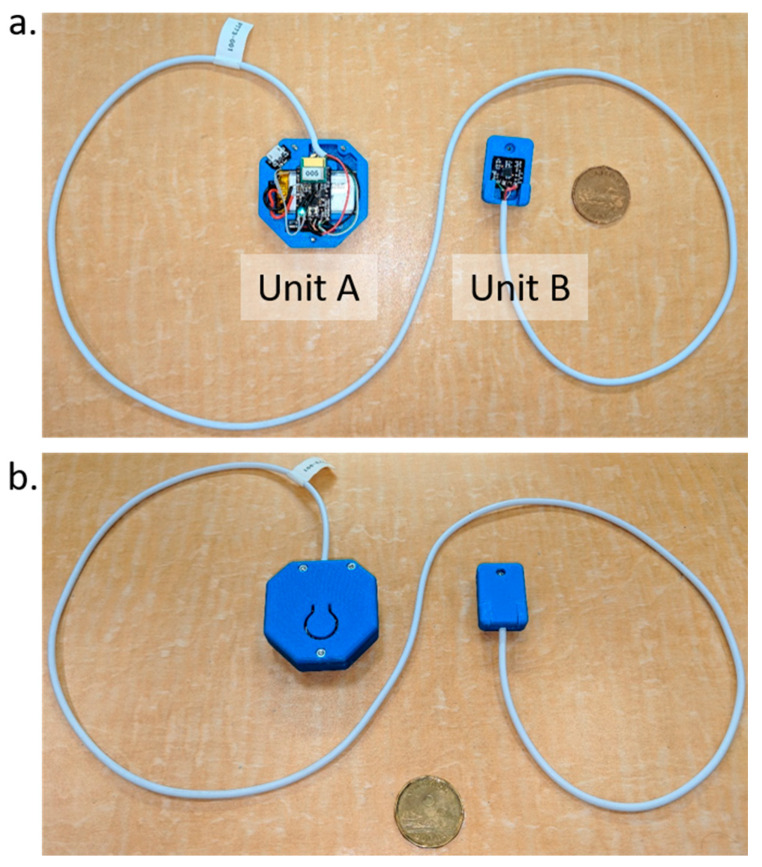
Wearable device for back posture monitoring, size compared to a Canadian dollar coin: (**a**) circuitry view, and (**b**) closed device.

The setup included a wearable device, a laptop, and a video camera. The wearable device is composed of two IMUs (LSM9DS1 from ST microelectronics^®^) called units *A* and *B*. A battery, a Cypress microcontroller (Cypress PSoC 4 BLE -CYBLE-214009-00), a Bluetooth Low-Energy (BLE) antenna (CY5677 CYSMARTTM BLE 4.2 USB DONGLE) for data transmission, and a haptic feedback module (Pager motor 4.6 × 12 mm W/Rotating Head 1.5 V) were integrated in the unit *A* case, close to the clip for the pants. Each pulse signal of the haptic feedback was 300 ms “On” and 200 ms “Off” controlled by pulse-width modulation (PWM). The entire haptic feedback length was about 1.5 s. The microcontroller on unit *A* was programmed to read the IMUs and calculate the relative pitch, roll, and yaw angles between the two units, and perform all the computations to trigger the haptic feedback. Unit *A* was placed at the L4 vertebrae level for all participants, while unit *B* was placed at the T5 vertebrae level. Figure 1 shows the wearable device with all the components. The laptop was used to collect the data by a BLE wireless connection to the device. The sample rate of the data was set to 50 Hz. The selected frequency was a compromise for having a high resolution with regards to human motion and high stability of the BLE wireless connection. To validate the movement against the data, we used a video camera that recorded the complete data collection. A customized Java^®^ interface (Java-Oracle Corporation, Santa Clara, CA, USA) was developed to synchronize, save, and label sections between the data and video. Furthermore, participants were provided with the tools to accomplish the required tasks. The aforementioned tools were a medicine ball of 11.5 kg with a 60 cm diameter, a chair, a blanket, and a bed with adjustable height.

#### 2.2.1. Experimental Tasks

Three tasks were selected for this study: T1—mock patient transfer, T2—mock boosting patient into the bed, and T3—mock “bed making”. These tasks were selected because of the associated high risk and frequency of use in the profession [4,10,11].

For T1, the participants were asked to transfer the medicine ball from the chair to the bed. The medicine ball was simulating the trunk of a patient and was selected to reduce the risk of injury during the experiment. The ball was easy to handle and was of low weight in comparison to the use of a mannequin for patient handling training or a human volunteer. The chair was positioned approximately 15 cm away from the bed. The participants were instructed to handle the ball pretending it was a patient, being careful not to drop the ball or hit corners or surfaces during the whole task. During this task, the participants were free to approach the chair freely. T2 entailed partially lifting the medicine ball and sliding it on the bed. The participants were instructed to always perform this task on the same side of the bed. Similar to T1, the participants were instructed to carefully lay the ball down. In the last task (T3), participants were required to place a blanket on the bed, mocking the actions of making a bed. The trunk movements were recorded along all the tasks with the device and the video camera. Table 1 describes the correct performance for each task and the risks associated if they were performed incorrectly.

#### 2.2.2. Ethical Considerations

The Office of Research Ethics at Simon Fraser University approved the study protocol, and all participants provided informed consent before participating. The objectives and voluntary nature of the study were explained to the participants. Confidentiality was assured, as no identifying information was collected during the study. All participants read and signed the consent form. Participants were recruited via social media, email, or in person following a script approved by the ethics office. The purpose of this script was to engage the participants without bias and avoid providing misleading information.

### 2.3. Experimental Procedures

We divided the study into four sessions. The first session (session zero) let the participants familiarize themselves with the device and setup. At the beginning of each session, an operator explained the study purpose, device features, tasks, and objects that were used for the tasks. During this phase, we used a script to provide consistent information to each of the participants.

**Figure 2:**
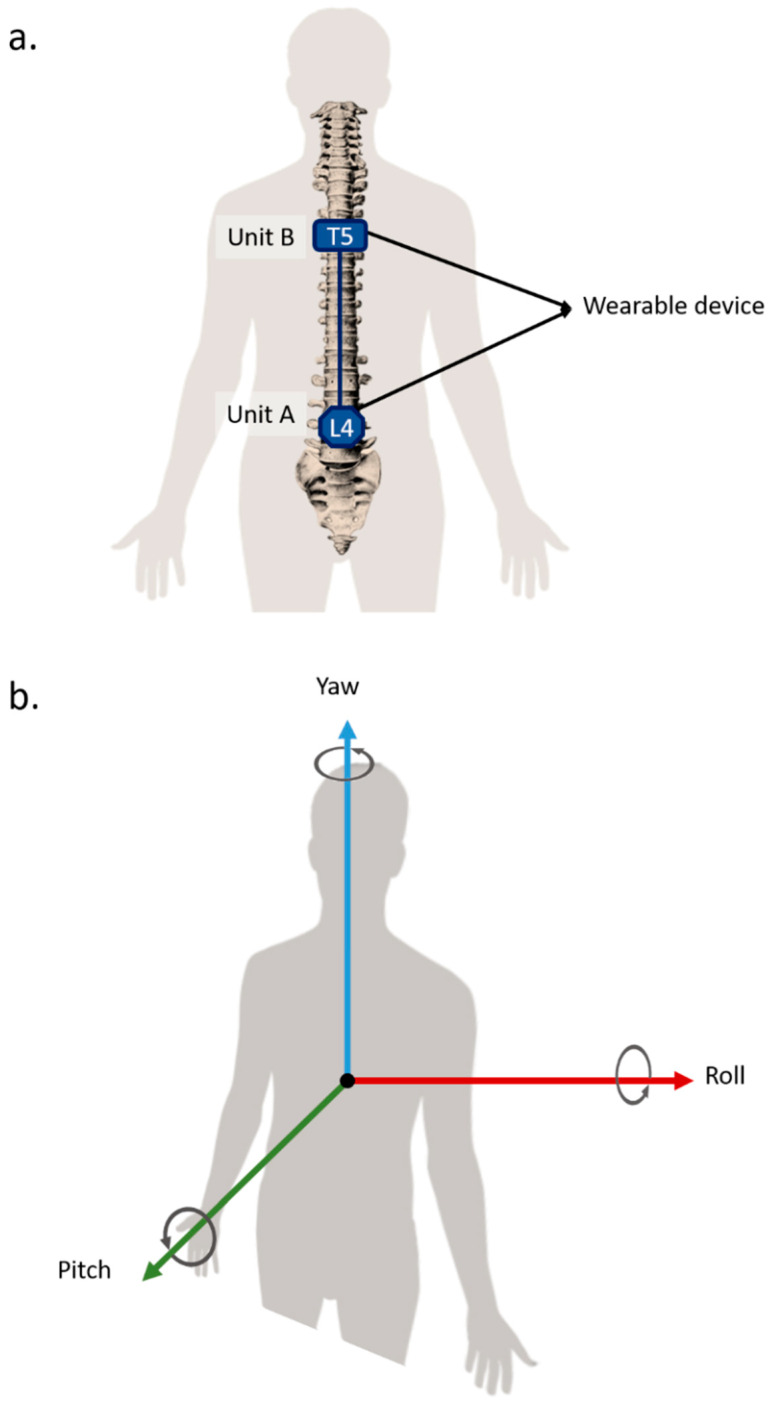
Wearable device and axis: (**a**) Wearable device positioned in T5 and L4 vertebrae. (**b**) Pitch, roll, and yaw axes definition used for the wearable device. These images are licensed under a Creative Commons Attribution-Share Alike license (CC BY—SA) [32,33].

All participants had the opportunity to inspect the device and ask questions. An operator was available to help them place the device on their back following the positioning illustrated in Figure 2a. The forward/backward bending correspond to rotations about the roll axis. The left/right lateral bending correspond to rotations about the pitch axis. Finally, the left/right twisting correspond to rotations about the yaw axis. A positive value corresponds to the movements forward bending, right lateral bending, and right twisting. The axis definitions used for the wearable device are shown in Figure 2b.

After the device was correctly positioned on the participant’s body, we began the calibration process. The calibration process was composed of five movements: neutral standing with a straight back, maximum forward bending, left and right bending, and sitting. These movements were meant to record the range of motion of each participant and to calibrate the feedback of the device on a fixed percentage. For this study, the device was programmed to provide vibration feedback only for back flexion (forward bending). The angles were calculated using the relative orientation of unit B in relation to unit A, where the upright posture corresponded to 0°. Additionally, the device vibrated when the participant performed a forward bending that exceeded the fixed percentage of the maximum flexion recorded during the calibration at session 0 (familiarization). The participants were asked to move freely and try to trigger the vibration with their movements to familiarize themselves with the haptic feedback.

It was explained to the participants that the haptic feedback (3 pulses of vibration) meant that in that exact instant, they were in an incorrect posture or performing a dangerous movement.

**Figure 3:**
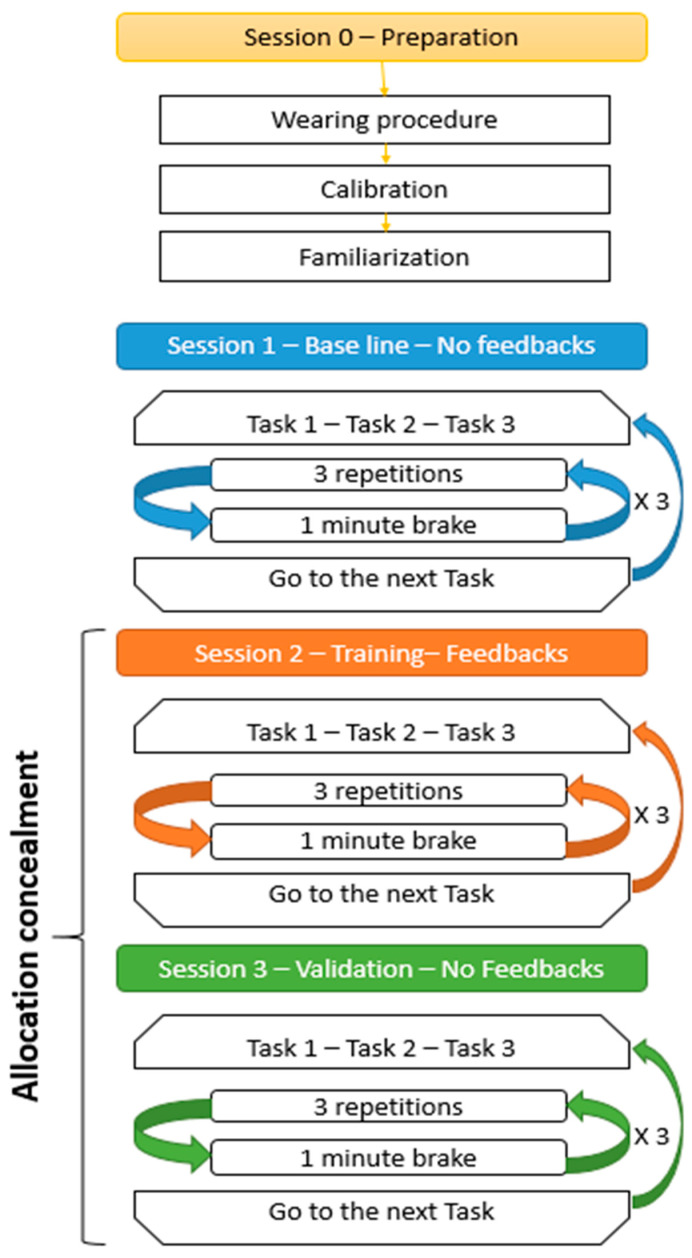
Experimental procedure: sessions and cycle structure.

For session one, participants were required to perform the three tasks (T1, T2, and T3). Each task was composed of three repetitions of the movement plus one minute of rest. The sequence of three repetitions plus one-minute rest was repeated three times. Once all the repetitions were completed, the participant moved on to the next task and started the cycle over. Figure 3 shows the sessions and cycle structure. The haptic feedback was turned off for all of session one, and the movements were recorded to establish a baseline for each participant.

For session two, the tasks, movements, and repetitions were the same as in session one; however, during session two, the haptic feedback was enabled. In this session, the participant was “trained” by the device.

Finally, in session three, the haptic feedback was turned off again. We adopted an allocation concealment technique to minimize the bias. The participants were not aware of the existence of session three, they were only instructed to repeat the tasks of session two.

### 2.4. Threshold Computation

The threshold is one of the key points of a device that has the scope to increase the awareness of bad postures/movements and correct the behavior of the users.

For this study, we chose to provide instant feedback any time the participant exceeds the threshold. To avoid false positives due to noise in the readings, the trigger was activated only if ten consecutive samples that exceed the threshold in value were collected. Meaning that the vibration was provided every time there was a transition from a safe posture to a dangerous posture, where the dangerous posture was maintained for more than 200 ms (data acquired at 50 Hz).

To define the threshold value, we referred to the ISO 11226:2000, where the trunk inclination angle (measured from the hip to the neck) is not acceptable under any conditions if it exceeds 60° [34]. Furthermore, biomechanical studies proved that a forward bending movement starts with a greater contribution to the motion at the lumbar vertebrae compared to the pelvis. However, at the late phase of the forward bending, the motion is predominantly at the pelvic area [35].

The lumbar-pelvic region has a maximum range of motion of approximately 110°, where 30% of the total range of motion is performed by the lumbar vertebrae [36]. Moreover, the average range of motion when flexing the thoracic vertebrae is approximately 26° [37]. Ideally, trunk flexion performed mainly by pelvis rotation with minimum work of the trunk and lumbar region is considered safe and well-performed [30].

Studies suggested that the spine should maintain a neutral curvature (thoracic kyphosis and lumbar lordosis) during sustained postures or while performing lifting actions [30]. Considering this statement, the principal focus of our device was to monitor flexion angles performed by the trunk and lumbar area. This was achieved by calculating the relative rotational angle between the thoracic and lumbar areas.

For this purpose, we computed our threshold starting from the value proposed by the ISO normative (60°) and calculated it as a percentage of the maximum flexion of the spine (140°), as studied in the literature [34,37]. The result of the computation was 42%. We use this percentage as a starter threshold to trigger the haptic feedback. Following the ISO recommendations, any movement or posture under any condition should not surpass that percentage. Furthermore, we considered the variability of the flexibility and range of motions for each participant by calculating the percentage from their maximum motions during flexion tasks. Therefore, the haptic feedback was activated when the participants reached 42% of their maximum flexion, as measured in session 0 (familiarization). In addition, the device was adjusting the threshold for each participant by considering the calibration movement and the movements performed during the session 0, computing 2 times the standard deviation during flexion tasks. Finally, the device calculates the boundaries and calibrates itself based on the calibration movement performed by the participant.

### 2.5. Data Analysis

The raw data of the participants’ movements during this study were collected by the wearable device at 50 Hz and transmitted via BLE to a laptop, where a Java Desktop App organized the data in Excel files (Microsoft Corporation, Microsoft Excel, Redmond, WA, USA). The data were analyzed using MATLAB (MathWorks, Natick, MA, USA). The data analysis mentioned in this section is post-processed, as the built-in data processing is proprietary. To minimize bias, the value of the threshold for each participant was hidden. Additionally, the type of task performed by the participant and the corresponding file for each participant was also hidden. Hence, it was necessary to create new parameters to define the participants’ performance.

**Figure 4:**
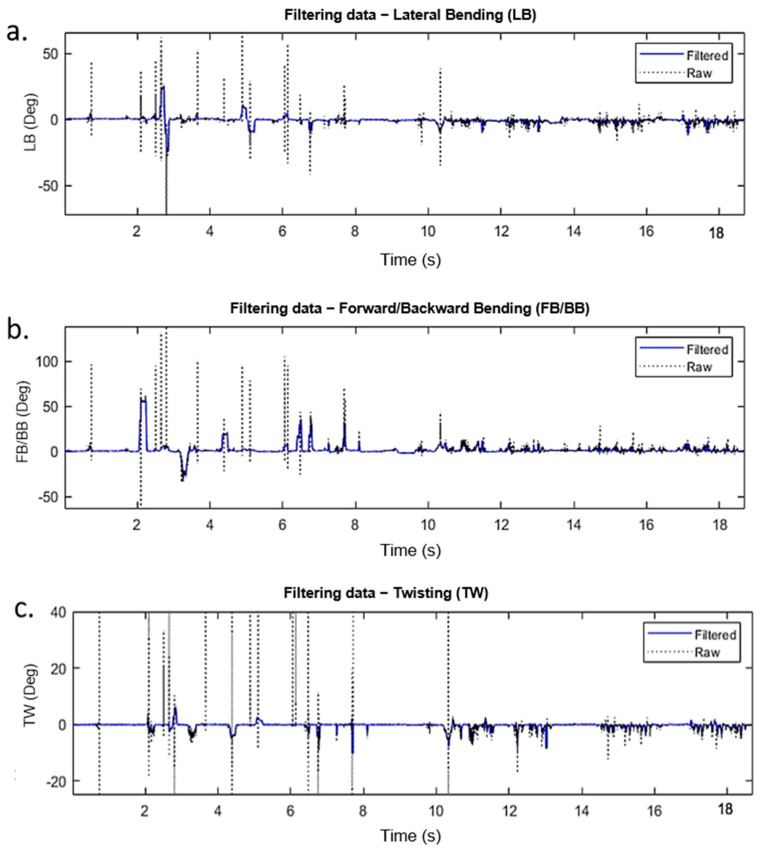
Raw vs. filtered data of a single participant using the *medfilt* function: (**a**) Lateral bending (LB), (**b**) forward/backward bending (FB/BB), and (**c**) twisting (TW).

The raw data obtained by the wearable device were first filtered without losing valuable information. To achieve this, we used the “*medfilt1*” MATLAB function. As described from the documentation online “This function replaces every point of a signal by the median of that point and a specified number of neighboring points. Accordingly, median filtering discards points that differ considerably from their surroundings”. For our application, the filter order was 15. This filtering gave us a reliable outcome with a high confidence level that the filtered points were not coming from noise or interferences. Figure 4 illustrates a sample of a raw dataset and the filtered data.

**Figure 5:**
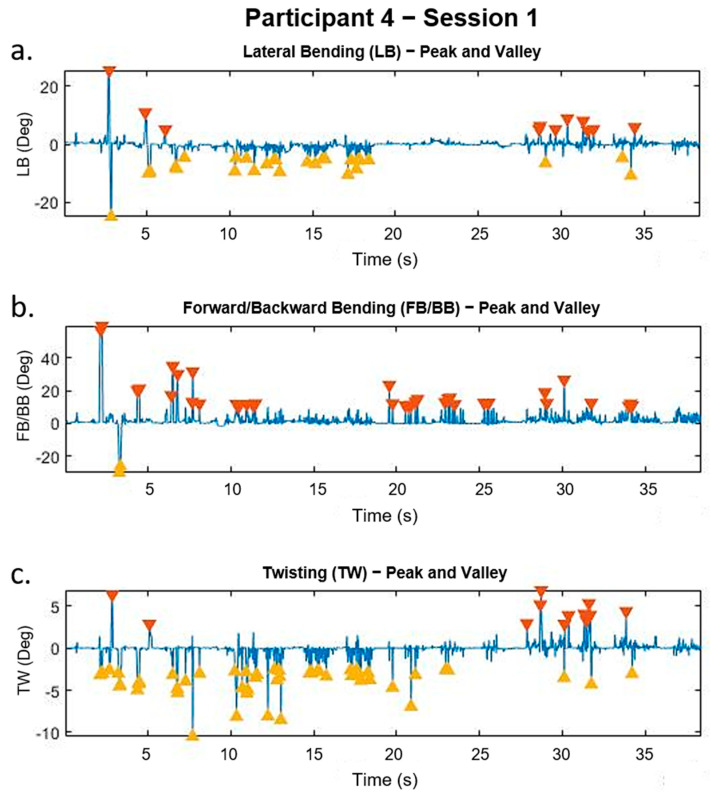
Peaks and valleys in the data of a single participant using the *findpeaks* function: (**a**) lateral bending (LB), (**b**) forward/backward bending (FB/BB), and (**c**) twisting (TW).

To define the performance of a participant, the MATLAB function “*findpeaks*” was used. This function can identify the peaks of a signal based on customizable parameters. We selected to identify peaks in the data that had a width greater than 10 points (200 ms) and a height greater than two times the standard deviation of the whole signal. The results of this function provide us a number (i.e., the number of peaks and valleys identified) that was considered as a parameter to evaluate the performances of the participants between the sessions. Figure 5 shows an example of the “*findpeaks*” function using the data of a single participant.

The performance and improvement of each participant were evaluated by comparing the standard deviation, maximum angle, and the total number of movements performed between the three sessions. Then, the average improvement for the combined participants was calculated.

## 3. Results

The data from two participants were corrupted and incomplete. Due to COVID-19, it was not possible to reschedule the data collection for these participants. We decided to remove these data to avoid incorrect interpretations of the general performance and improvement of the participants. The data of the remaining 7 participants (age: 26.0 ± 5.6 years, height: 1.57 ± 0.06 m, weight: 56.78 ± 7.15 kg) were used for the analysis.

Table 2 presents the maximum angle of the peaks recorded by each participant when performing forward bending (FB), right lateral bending (LB), and right twisting (TW). Table 3 presents the maximum angle of the valleys recorded by each participant when performing backward bending (BB), left lateral bending (LB), and left twisting (TW). Each table includes the data recorded from the three sessions (baseline, training, and validation) for right/left lateral bending, forward/backward bending, and right/left twisting. Finally, in this study, we considered the participant upright posture as angle zero.

**Figure 6:**
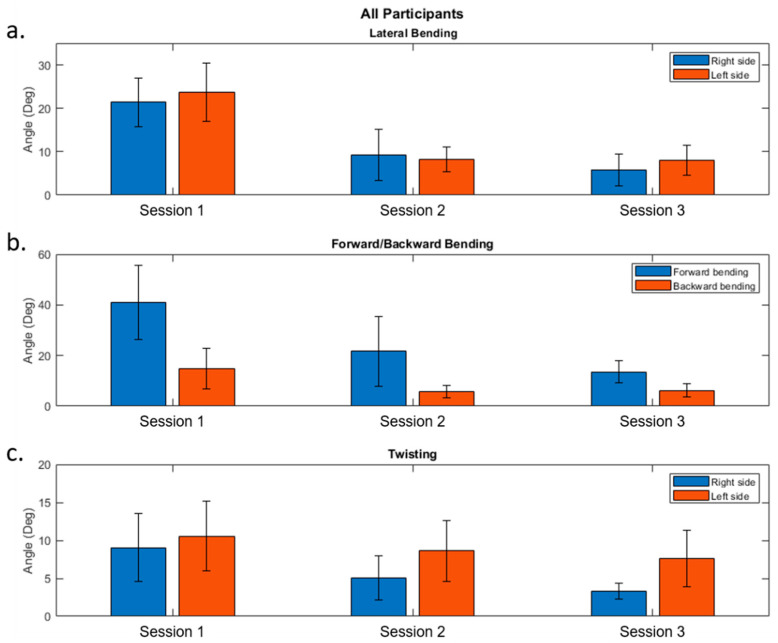
Performance of all the participants for sessions 1, 2, and 3. (**a**) Lateral bending, (**b**) forward/backward bending, and (**c**) twisting.

The combined performance of all participants is illustrated in Figure 6. This figure contains three bar graphs that illustrate the results for the FB/BB, LB, and TW. Each bar graph shows the results from sessions 1, 2, and 3. Furthermore, the blue, orange, and yellow bars represent the values for maximum angle (FB, right LB, and right TW), maximum angle (FB, left LB, and left TW), and the standard deviation, respectively.

The improvement (in percentage values) of the participants was calculated and averaged to evaluate the general improvement in this study. The improvement was obtained by calculating the percentage of the variables: maximum FB, right LB, right TW, BB, left LB, left TW, and standard deviation of all the sessions. We calculated the improvement percentage for session 1–session 2, session 2–session 3, and session 1–session 3, shown in Table 4, Table 5 and Table 6, respectively. In Table 4 and Table 6, we considered the values of session 1 as 100 percent, whereas in Table 5, the values from session 2 were considered as 100 percent. The positive values in Table 4, Table 5 and Table 6 represent an improvement, while the negatives values show a decline.

The positive percentage values in Table 4, Table 5 and Table 6 represent a decrease in the value from one session to the other. A positive percentage value is considered an improvement since the participants are avoiding dangerous flexion angles.

**Figure 7:**
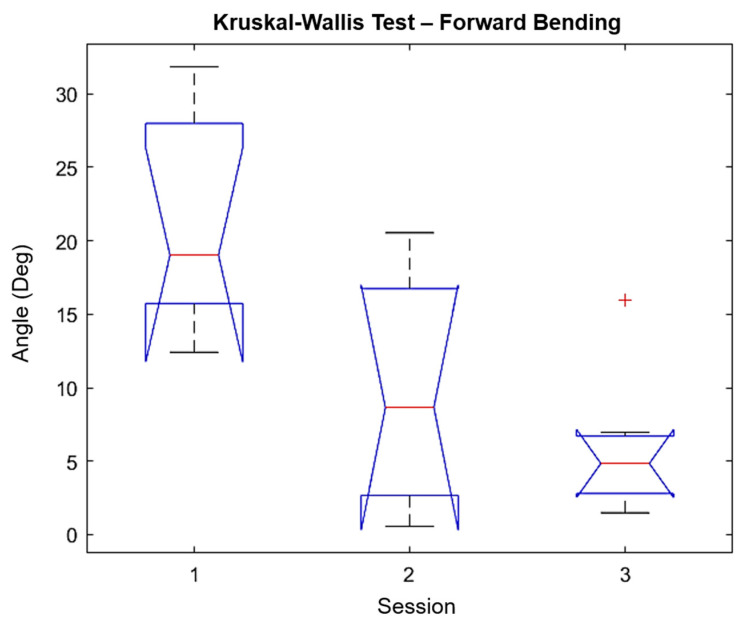
Kruskal–Wallis test for forward bending. The notch in session 3 does not have overlap with the notch in session 1, which means that it has a different median (Q2/50th Percentile) at the 5% significance level. Q1 has a decreasing trend over the 3 sessions. Q3 is not decreasing consistently along the 3 sessions.

**Figure 8:**
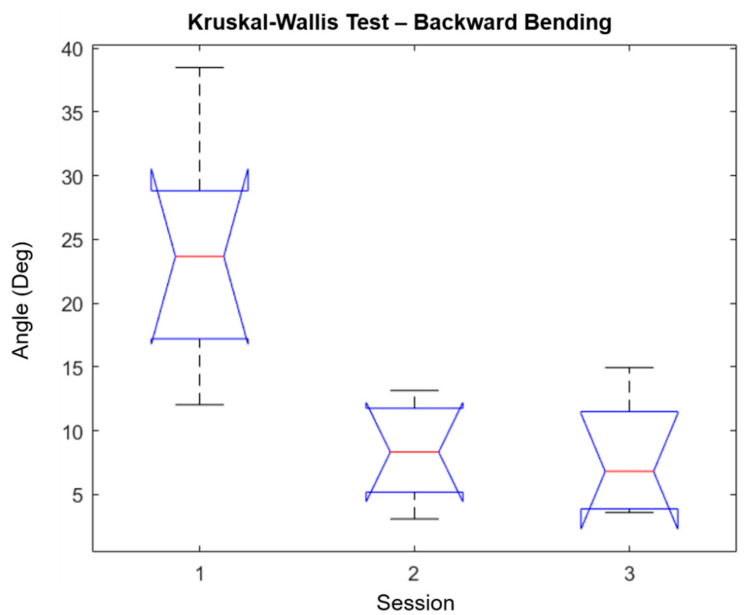
Kruskal–Wallis test for backward bending. The notch in sessions 2 and 3 does not overlap with the notch in session 1, which means that they have different medians (Q2/50th Percentile) at the 5% significance level. Q1 is decreasing in sessions 2 and 3 in comparison to session 1. Q3 has a decreasing trend over the three sessions.

**Figure 9:**
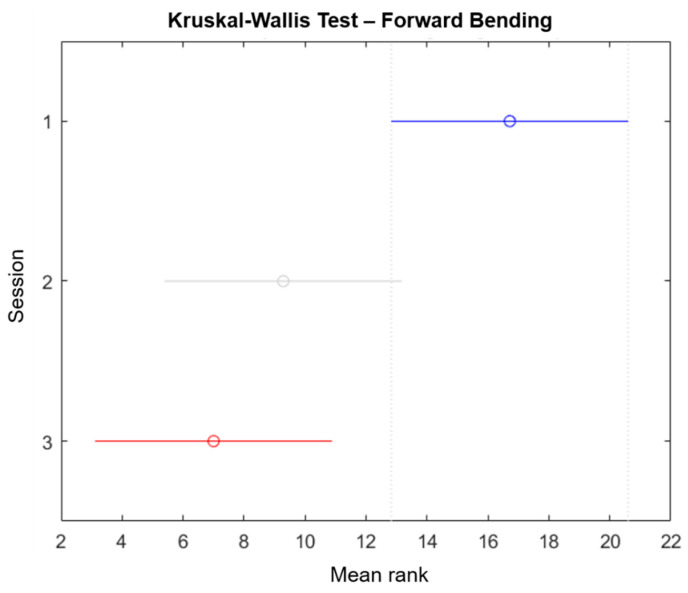
“Multicompare” from the Kruskal–Wallis test for forward bending, where the mean ranks of sessions 1 and 3 are significantly different.

**Figure 10:**
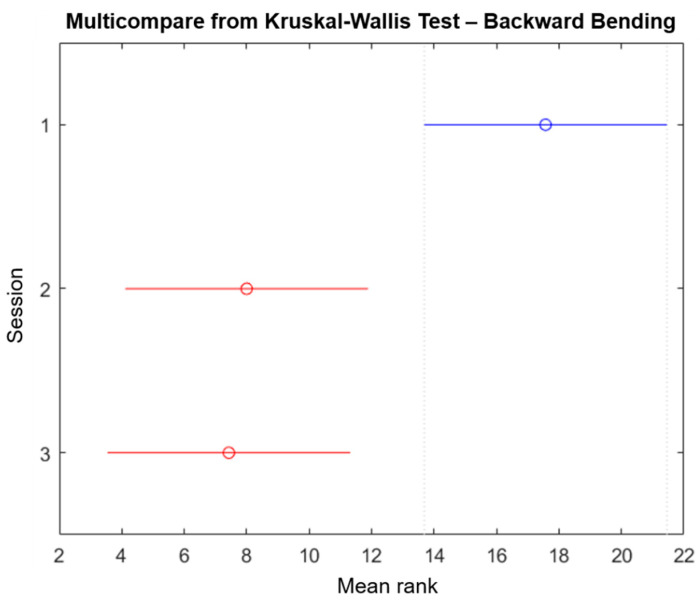
“Multicompare” from the Kruskal–Wallis test for backward bending, where sessions 2 and 3 are significantly different from session 1.

We performed a Kruskal–Wallis test using the Matlab function “*kruskalwallis”* to evaluate the significance and statistic of the sessions performed in this study (only the forward/backward bending is reported). The Kruskal–Wallis test is a non-parametric approach to the one-way ANOVA that compares the medians of the group data to determine if the samples come from the same population, where the chi-square is the H-statistic of the Kruskal–Wallis test and the *p*-value measures the significance of the chi-square statistic. The Kruskal–Wallis test for forward bending is shown in Figure 7, while the results for backward bending are shown in Figure 8. The *p*-values and chi-square reported for forward bending movements were 0.0092 and 9.38 respectively, while the *p*-values and chi-square reported for backward bending movements were 0.0027 and 11.81, respectively. Additionally, the mean ranks for the forward bending and backward bending are shown in Table 7. In both cases, the degrees of freedom were 2. Finally, the “multicompare” function results for forward bending and backward bending are shown in Figure 9 and Figure 10, respectively. From these results, it is possible to observe that session 1 and session 3 were significantly different. However, only for backward bending were sessions 2 and 3 significantly different from session 1.

## 4. Discussion

In this paper, we introduced a wearable device for the prevention of LBP in nurses. The device was comprised of two IMUs that were attached to the participants at the level of T5 and L4. We also reported the impact of receiving haptic feedback when a bad posture was performed during three specific movements (mock patient transfer, mock boosting a patient in bed, and reaching task).

The device was fabricated to be wireless and unobtrusive so the participants could freely perform their activities. Previous articles have presented different approaches to monitoring back movements, such as textile sensors, accelerometers, IMUs, goniometers, and strain gauges [7,22,38,39,40,41]. However, not all the prototypes provide haptic feedback, which translates to poor management of postural improvement. Furthermore, there are few devices available in the market that have haptic feedback [23,24,25,26,27,28]. Unfortunately, the general disadvantage of these devices is the limitation of movements that can be monitored.

During session zero, the participants were able to familiarize themselves with the device. The participants did not report being uncomfortable when wearing the device. However, some participants were not able to feel the vibration used for the haptic feedback. This issue was addressed before starting the test by increasing the vibration intensity. We experimentally tested that the perception of the vibration on the skin was stronger with pulses than with one single long vibration of 1.5 s. Moreover, when the participant was wearing loose pants/garments, the haptic feedback was not perceived correctly. Therefore, we asked the participants to tighten their pants or wear a tighter garment. We found it was particularly important that the participants felt comfortable to reduce the laboratory environment’s bias.

We noticed during session 2 (Training) that as soon as the participants felt the haptic feedback, they immediately tried to correct their posture to stop the vibration. This is not surprising since they were informed of the meaning of the haptic vibration at the beginning of the test.

The average maximum peak angles performed during session one were 21.39, 41.09, and 9.06 degrees for right LB, FB, and right TW, respectively. While for session two, they were 9.29, 21.66, and 5.06 degrees for right LB, FB, and right TW, respectively. We observed a decrease in the maximum peak angles performed by the participants when comparing the baseline session with the training session. The participants were able to improve their postures when performing the three different movements. For right lateral bending, we found that the participants reduced their bending angles by 58.9%. For forward bending, there was a reduction of 48.3%. Finally, for right twisting, the participants presented a reduction of 37.1%. The average maximum valley angles performed during session one for left LB, BB, and left TW were 23.68, 14.78, and 10.55 respectively, while the values for session two were 8.20, 5.72, and 8.62 for left LB, BB, and left TW, respectively. As before, there was an important reduction of the bending angles from the baseline to the training session. The average percentages of reduction for left LB, BB, and left TW were 65.1%, 44.6%, and 7.3%, respectively.

The results from comparing the improvement from the baseline session to the training session proved the impact of the haptic feedback. During session 2, the participants were trying to avoid triggering the vibration system by reducing the back bending in the three axes. Avoiding awkward postures is a common technique to prevent LBP, for example, Nelson et al. [13] reported that nurses spend around 30% of their time performing forward bending or twisting. Furthermore, the impact that the haptic feedback had on our study aligns with the findings of Demircan [42], where he demonstrated that providing haptic feedback in real-time promotes motor learning.

Although there was a clear improvement in the participants’ posture between sessions 1 and 2, we still needed to investigate if the participants relied on the feedback to correct their posture or if they were learning to avoid dangerous postures. Therefore, we compared the results from session two to session three and session one to session three. It was important to remember that during session three, the feedback of the wearable device was set to off.

The average maximum peak angle during session three was 5.88, 13.59, and 3.29 degrees for right LB, FB, and right TW, respectively. These data show that even though the feedback was off, there was still a reduction of 11.1% and 17.0% for the FB and right TW, respectively. However, there was a worsening of 26.3% in the right LB. This average worsening was the result of a single participant that started from a very low values and had an increase in bending angles of more than 360% (participant 5, shown in Table 2). However, if we considered the improvement on the average angles instead of the average improvements, we still obtained a general reduction of bending angles of 36.5% from training (session 2) to validation (session 3). The average maximum valley angle during session 3 was 8.00, 6.22, and 7.59 degrees for left LB, BB, and left TW, respectively. When compared with session 2, there was an increase of 4.4% and 14.4% for left LB and BB respectively, while for the left TW, there was a reduction of 2.5%. Nevertheless, when the values for left LB and BB from session 3 were compared with session 1, there was still a reduction of 65.8% and 42.6%, respectively.

The results shown in Table 4 and Table 6 confirm that participants learn to adopt better postures when performing the different movements. Moreover, Figure 9 and Figure 10 corroborate this conclusion by showing that session 1 was significantly different to session 3. Generally, the posture of all participants improved even after the haptic feedback was turned off. These results align with the finding reported by Jaromi et al. [4], where the participants were able to reduce their LBP by improving their body posture after receiving education and ergonomic skills. The only parameter that showed a lower improvement percentage in Table 6 when compared with Table 4 was the maximum valley angle for backward bending, with 42.8%. However, the results from session 2 and session 3 showed that the maximum angle of (filtered) forward bending of all participants was lower than the 60° limit of trunk inclination set by the ISO 11226:2000 [34].

These results showed that, in general, the participants were able to learn to avoid dangerous postures while wearing the device. However, it is important to highlight that the sessions were performed consecutively with short periods of rest in between. Therefore, for future studies, the impact of haptic feedback in the long term should be evaluated.

One limitation of this study was that for T1 (mocking patient transfer), we used a medicine ball instead of a person or a mannequin. We purposely chose the medicine ball to avoid injuries during our test. However, this modification may not have represented the patient transferring movements accurately. Another limitation was the number of participants enrolled in the study. The occurrence of COVID-19 disrupted our recruiting scheduling and avoided the continuation of the data collection. Despite the data showing promising results for this device, having more participants could improve the quality of the statistics. Additionally, the movements that we selected did not cover all of the activities that a nurse performs during their working day. Hence, it is necessary to evaluate the performance of the device inside a hospital.

## 5. Conclusions

The haptic feedback was shown to have an important impact on the participants’ posture. Moreover, the participants tried to immediately correct their posture when the device gave them haptic feedback. Finally, this study shows that the participants were able to learn to avoid dangerous postures after the training session.

## Figures and Tables

**Table 1:** Performance for each task.

Movement Type	Correct Performance Instructions [30]	Potential Risks Associated to Movement
T1—Mock patient transfer	The participant will raise the bed to an appropriate level, and bend legs to lift the ball off the bed. Hold the ball close to the chest while pivoting. Then, bend legs to place the ball on the chair. Finally, the participant returns the ball to the starting place and returns to the starting position. The chair was always positioned on the same side of the bed.	Bending forward to lift the medicine ball without bending the knees may cause dangerous pressure in the lumbar vertebrae [31]. Lifting the medicine ball using only the arms and maintaining the ball away from the chest may cause poor control and balance, and result in dangerous back angle or high pressure in the lumbar vertebrae and shoulders.
T2—Mock boosting patient in bed	Stand up with feet shoulder-width apart and hold the ball. Shift weight from the back leg to the front leg to shift the patient from the lower part of the bed to the upper part. Always on the same side of the bed. Finally, the participant returns the ball to the starting place and returns to the starting position.	If performed incorrectly, the back will be used to facilitate the movement rather than the legs. This may cause dangerous pressure in the lumbar vertebrae [31].
T3—Reaching tasks	Ideally, subject will walk around the bed to extend the sheet across the bed, rather than extending the body across the bed to reach the far corners. Finally, the participant returns the sheet to the starting place and returns to the starting position.	Reaching across the bed to secure the sheet may lead to dangerous back angles.

**Table 2:** Maximum peak values in degrees.

	Session 1 (Baseline)			Session 2 (Training)			Session 3 (Validation)		
No.	Right LB	FB	Right TW	Right LB	FB	Right TW	Right LB	FB	Right TW
1	12.41	18.87	1.96	3.69	11.99	2.00	4.84	13.20	2.27
2	25.21	59.23	6.56	10.09	17.56	6.09	3.36	13.26	2.20
3	16.99	36.76	7.93	2.35	23.07	2.36	1.48	11.97	2.57
4	31.84	62.88	17.77	8.66	61.23	4.82	6.96	17.48	2.87
5	15.31	20.77	5.00	0.56	5.81	2.10	2.61	10.46	2.43
6	19.03	60.30	7.31	20.54	16.62	5.11	5.91	23.39	5.47
7	28.92	28.83	16.89	18.95	15.35	12.97	15.99	5.40	5.22
AVG	21.39	41.09	9.06	9.26	21.66	5.06	5.88	13.59	3.29

**Table 3:** Maximum valley values in degrees.

	Session 1 (Baseline)			Session 2 (Training)			Session 3 (Validation)		
No.	Left LB	BB	Left TW	Left LB	BB	Left TW	Left LB	BB	Left TW
1	12.04	13.53	2.37	3.09	3.48	1.82	3.71	3.89	0.94
2	24.66	29.78	10.18	10.02	2.81	13.61	14.95	4.19	14.42
3	23.66	9.37	9.44	13.15	4.36	15.63	4.36	5.33	4.74
4	30.19	29.29	10.60	5.13	7.63	9.05	6.81	10.73	6.20
5	38.47	10.34	14.05	12.33	11.75	11.78	10.85	6.68	14.21
6	16.05	4.31	21.47	5.38	2.42	3.43	3.58	1.86	6.04
7	20.70	6.87	5.74	8.33	7.58	5.03	11.71	10.85	6.61
AVG	23.68	14.78	10.55	8.20	5.72	8.62	8.00	6.22	7.59

**Table 4:** Improvement percentage session 1–session 2.

	LB (deg)	FB/BB (deg)	TW (deg)
maximum peak values	58.9%	48.3%	37.1%
maximum valley values	65.1%	44.6%	7.3%
standard deviation	45.7%	52.0%	21.2%

**Table 5:** Improvement percentage session 2–session 3.

	LB (deg)	FB/BB (deg)	TW (deg)
maximum peak values	−26.3%	11.1%	17.0%
maximum valley values	−4.4%	−14.4%	2.3%
standard deviation	11.3%	6.0%	9.8%

**Table 6:** Improvement percentage session 1–session 3.

	LB (deg)	FB/BB (deg)	TW (deg)
maximum peak values	73.4%	62.8%	49.7%
maximum valley values	65.8%	42.8%	23.7%
standard deviation	51.5%	55.7%	31.6%

**Table 7:** Mean ranks for forward/backward bending in degrees.

	Session 1	Session 2	Session 3
maximum peak values	16.71	9.29	7
maximum valley values	17.57	8.00	7.43

## Data Availability

Data sharing not applicable.
